# Wound-induced transcriptional dynamics in rice

**DOI:** 10.1007/s44297-025-00055-2

**Published:** 2025-07-01

**Authors:** Yumeng Chen, Gaochen Jin, Jing Lu, Yonggen Lou, Guillermo H. Jiménez-Alemán, Ran Li

**Affiliations:** 1 State Key Laboratory of Rice Biology and Breeding, Zhejiang Key Laboratory of Biology and Ecological Regulation of Crop Pathogens and Insects, Institute of Insect Sciences, Zhejiang University, Hangzhou, China; 2 Department of Chemistry and Environmental Science, New Jersey Institute of Technology, University Heights, Newark, NJ 07102 USA

**Keywords:** Wound, Rice, Transcriptome, Jasmonate, Leaf folder

## Abstract

Herbivore attacks or some pathogen infections typically begin with wounding of plant tissues, yet the transcriptional dynamics of wound-induced responses in the monocot rice remain incompletely understood. Here, we conducted a time-series transcriptome analysis of mechanically wounded rice leaves. Temporal expression patterns were observed even in untreated plants, particularly at dusk. To identify differentially expressed genes (DEGs), we compared wounded and unwounded (control) plants at corresponding time points. Jasmonate-related genes, including 18 biosynthetic and 13 catabolic genes, were significantly up-regulated. Consistently, jasmonic acid (JA) and jasmonoyl-L-isoleucine were rapidly accumulated in wounded tissues within 1 h, followed by JA catabolites after 3 h. Weighted gene co-expression network analysis of upregulated DEGs revealed early- and late-responsive gene modules. Early-expressed genes included putative regulators such as transcription factors, kinases, abscisic acid pathway components, and small peptide-coding genes, while late-responsive genes were primarily involved in specialized metabolite biosynthesis (e.g., phenylpropanoids). Targeted metabolomic analysis showed that most phenolamides were highly accumulated in wounded leaves, whereas flavonoid levels were decreased, likely due to altered metabolic flux in the phenylpropanoid pathway. Comparative analysis of leaf folder (LF) larvae- and wound-induced transcriptomes revealed that only 42% of wound-upregulated DEGs responded to LF feeding, suggesting that LF larvae partially suppress wound responses in rice. This study provides a valuable resource for understanding wound-associated biotic stress responses in rice.

**Supplementary Information:**

The online version contains supplementary material available at 10.1007/s44297-025-00055-2.

## Introduction

Plants frequently encounter various biotic stresses in their natural habitats, many of which cause wounds on plant tissues, such as herbivore attacks or fungal/nematode infections. Upon wounding, damage-associated molecular patterns (DAMPs), such as plant elicitor peptides (Peps) are released from damaged cells and recognized by membrane receptors [1]. This perception triggers intracellular signaling cascades that activate the expression of wound-responsive genes [1]. For instance, Peps, consisting of 23 amino acids, are derived from precursor proteins known as PROPEPs [2], whose transcription is induced by wounding. These peptides are recognized by leucine-rich repeat (LRR) receptor-like kinases, termed Pep receptors (PEPRs), in *Arabidopsis* and rice [3–5]. Peps-mediated signaling enhances plant resistance to fungus and herbivores by promoting the biosynthesis of defense-related hormones such as jasmonic acid (JA) [6–8].

Protein phosphorylation occurs within minutes after wounding, highlighting the role of protein kinases in wound-induced signaling transduction [5, 9, 10]. Plant mitogen-activated protein kinase (MAPK) cascades are known to regulate wound responses [9, 10]. In *Arabidopsis*, the kinase activity of AtMPK2 could be activated as early as 30 min post-wounding, with its upstream cascade gene, *AtMKKK14*, showing significant upregulation within 15 min. Mutations in *AtMKK3* and *AtMKKK14* impair the kinase activity of AtMPK2 [11]. Additionally, the kinase activities of MPK3, MPK4, and MPK6 are also induced by wounding [12, 13], while overexpression of the PP2C-type phosphatase *AP2C1* disrupts wound signaling by dephosphorylating and inactivating MPK4 and MPK6 [14].

Phytohormones play a central role in wound responses with JA being the most well-known wound-related hormone. JA is essential for both plant defense and wound regeneration or healing [15, 16]. Its biosynthesis occurs in response to several factors, including wounding, and begins with α-linolenic acid hydroperoxidation (LOX enzyme mediated) in the chloroplast and continue with the enzymatic conversion by an allene oxide synthase (AOS), and allene oxide cyclase (AOC) enzymes, leading to the formation of 12-oxo-phytodienoic acid (OPDA). OPDA is then converted into JA through both OPDA reductase 3 (OPR3)-dependent and -independent pathways in the peroxisome [17–19]. In the cytosol, JA is further metabolized into various derivatives, among which jasmonyl-L-isoleucine (JA-Ile) is the most bioactive form in vascular plants [20]. JA-Ile binds to its coreceptors, which results in degradation of the JAZ family of repressors and the consequent activation of JA-responsive transcription factors such as MYC2 [21–25]. JA and JA-Ile can also be further metabolized through hydroxylation, glycosylation, and methylation [18]. Disrupting JA biosynthesis or perception abolishes a large part of wound responses in plants [26–28]. Beyond JA, other phytohormones also contribute to wound responses. The JA-auxin interaction is critical for de novo root regeneration, as wound-induced JA activates the ERF109 transcription factor, which upregulates the expression of *ANTHRANILATE SYNTHASE 1* (*ASA1*) to promote auxin biosynthesis and root regeneration [15, 29]. The induced JA can also promote abscisic acid (ABA) biosynthesis through MYC2, resulting in elevated ABA levels that sustain the expression of *RAP2.6*, ensuring lignin biosynthesis at wound sites and promoting wound healing [30].

Transcriptome analysis has been widely employed to gain a comprehensive understanding of plant wound responses. However, most studies have only collected samples at one or two time-points after treatment, limiting their ability to capture the dynamic changes in gene expression over time [31–33]. A study in the tea plant collected samples at four different time points after wounding, using 0 h (no wounding) as the control [34]. A more detailed analysis was conducted in *Lolium temulentum*, which included five time points with associated controls at each time point to elucidate the complete temporal wound responses [35]. Rice (*Oryza sativa* L.) is one of the most important staple crops and serves as a model monocot species. However, the global transcriptional dynamics following wounding remain poorly understood in rice. In paddy fields, herbivory and pathogenic infections can lead to significant yield losses. Notorious insect pests such as brown planthopper (BPH, *Nilaparavata lugens*) and leaf folder (LF, *Cnaphalocrocis medinalis*), along with the blast fungus (*Magnaporthe oryzae*), are known to cause wounds on rice tissues. In this study, we comprehensively investigated the wound-induced transcriptional dynamics in rice leaves by a time-series RNA-seq analysis. To capture the temporal expression profiles of wound-responsive genes, we collected leaf samples at three early (0.25, 0.5, and 1 h) and three late (3, 8, and 24 h) time points after wounding, with untreated plants at each time point serving as controls to exclude the influence of temporal gene expression. Differentially expressed genes (DEGs) were identified by comparing wounded with the respective (same age, unwounded) control samples. We focused on wound-induced up-regulated DEGs, which were further divided into distinct co-expression modules using weighted gene co-expression network analysis (WGCNA). Putative regulator and downstream responsive genes were identified by gene ontology (GO) enrichment analysis of early and late-expressed module genes, respectively. Moreover, we quantified wound-related phytohormones and specialized metabolites to validate the transcriptome data. Lastly, by comparing the wound-induced transcriptome with the transcriptome generated upon LF larvae feeding, we identified common and specific herbivore-elicited wound responses. Our results revealed comprehensive wound-induced transcriptional dynamics in rice, including the activation of JA metabolism and signaling, early wound-responsive transcription factors, kinases, peptides, ABA biosynthesis, and late wound-responsive specialized metabolites biosynthesis. Notably, LF feeding partially suppressed certain wound responses, such as the ABA-mediated signaling pathway.

## Results

### Wounding and time of the day induce significant transcriptional changes in rice

**Fig. 1:**
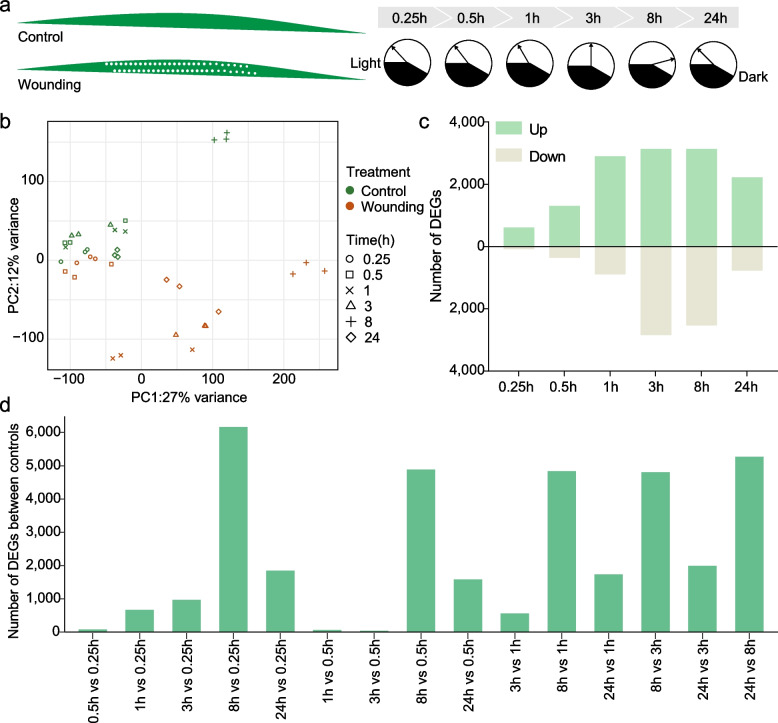
Experimental design and wound-induced time-series transcriptional changes in rice leaves. **a** Schematic diagram of the sample preparation and sampling. Rice leaves were wounded by rolling a fabric pattern wheel and harvested at six time points (0.25, 0.5, 1, 3, 8, and 24 h). Leaves from untreated plants at each time point served as controls. Three biological replicates were used for RNA-seq analysis. **b** Principal component analysis (PCA) of RNA-seq data of control and wound-treated plants. **c** Differentially expressed genes (DEGs) in wound-treated plants compared with control plants, with absolute |FC|> 2 and FDR value < 0.05 as cutoffs. **d** Differentially expressed genes (DEGs) between control plants at different points, with absolute |FC|> 2 and FDR value < 0.05 as cutoffs

To comprehensively evaluate wound-induced transcriptional dynamics in rice, we performed a time-series transcriptome analysis of rice leaves following mechanical wounding using a fabric pattern wheel. Leaf samples were collected at 0.25, 0.5, 1, 3, 8, and 24 h post wounding (hpw), with leaves from untreated plants at each time point serving as controls (Fig. 1a). Principal component analysis (PCA) of all transcripts clearly distinguished wounded samples and controls, indicating that wounding caused a large transcriptional reprogramming (Fig. 1b; Table S1). Interestingly, control samples collected at different time points were also separated, particularly those collected at dusk (8 h), suggesting that temporal gene expression patterns occur even in untreated plants. Thus, we screened the differentially expressed genes (DEGs) by comparing wounded and control samples at each time point (absolute fold-change (|FC|) > 2, false discovery rate (FDR < 0.05)) (Fig. 1c). Wounding induced a rapid transcriptional response, with 607 upregulated DEGs identified within 0.25 h. The number of DEGs increased over time, peaking at some point between 3 and 8 hpw. To evaluate the temporal transcriptional changes, we analyzed the DEGs among control samples collected at different time points. Consistent with PCA results, gene expression at 8 h significantly differed from other time points (Fig. 1d). Additional differences were observed in other comparisons, highlighting dynamic gene expression changes even in untreated plants.

### Wounding activates the JA signaling pathway but suppresses photosynthesis

**Fig. 2:**
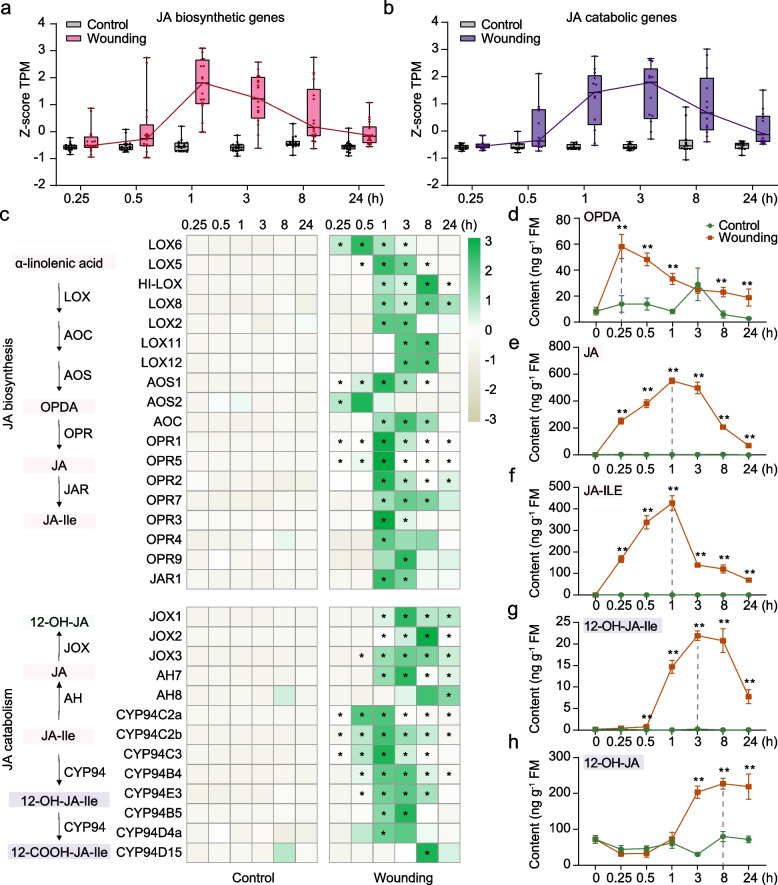
JA signaling pathway upon wounding treatment. **a-b** Box plots showing the total relative expression levels of JA biosynthetic genes (n = 18) (a) and JA catabolic genes (*n* = 13) (**b**) upon wounding treatment in RNA-seq data. In each box plot, the horizontal bar in the box indicates the median value. The upper and lower hinges of each box indicate the 75% and 25% ranges of the reported values, respectively. **c** Relative transcript levels of JA pathway genes in wounded and control leaves. Numbers in the color key indicate the row z-score of the TPM value in RNA-seq data. Asterisks indicate significant differences between different treatments at each time point (*, FC > 2 and FDR < 0.05). **d-h** Mean concentrations (± SE, n = 4–5) of OPDA (d), JA (e), JA-Ile (f), 12-hydroxy-JA-Ile (12-OH-JA-Ile) (g), 12-hydroxy-JA (12-OH-JA) (**h**) in rice leaves. The peak value of each compound at different time points was highlighted by the vertical dashed line. Asterisks indicate significant differences in wounded leaves compared with control leaves at each time point (*, *P* < 0.05; **, *P* < 0.01; Student’s t test)

The phytohormone JA is a well-established regulator of wound responses in plants [15, 27, 28]. In our analysis, we identified 18 known or putative JA biosynthetic genes and 13 JA catabolic genes among the wound-induced upregulated DEGs (Fig. 2a-c) [36, 37]. The relative total expression levels of JA biosynthetic DEGs peaked at 1 hpw (Fig. 2a), while JA catabolic DEGs peak at 3 hpw (Fig. 2b). Consistent with the gene expression, levels of the JA precursor 12-oxophytodienoic acid (OPDA), which peaked as early as 0.25 hpw (Fig. 2d). JA and JA-Ile began accumulating at 0.25 hpw and peaked at 1 hpw (Fig. 2e and f). In contrast, the accumulation of JA catabolites, 12-hydroxy-JA and 12-hydroxy-JA-Ile, was delayed, peaking at 8 hpw and 3 hpw, respectively (Fig. 2g and h). These results suggest that JA signaling is a conserved and rapid wound response in rice.

GO analysis showed that wound-induced down-regulated DEGs were significantly enriched in photosynthesis-related GO terms (Fig. S1a). The relative total expression levels of photosynthesis-related DEGs were decreased at 1, 3, and 8 h post-wounding (Fig. S1b), such as the light-harvesting complex II (*LHCB5*) and magnesium-chelatase ChlD (*ChlD*) protein-coding genes (Fig. S1c), suggesting that wounding may impair photosynthetic efficiency, thereby slowing plant growth.

### Weighted gene co-expression network analysis identified early and late wound-induced up-regulated gene modules

**Fig. 3:**
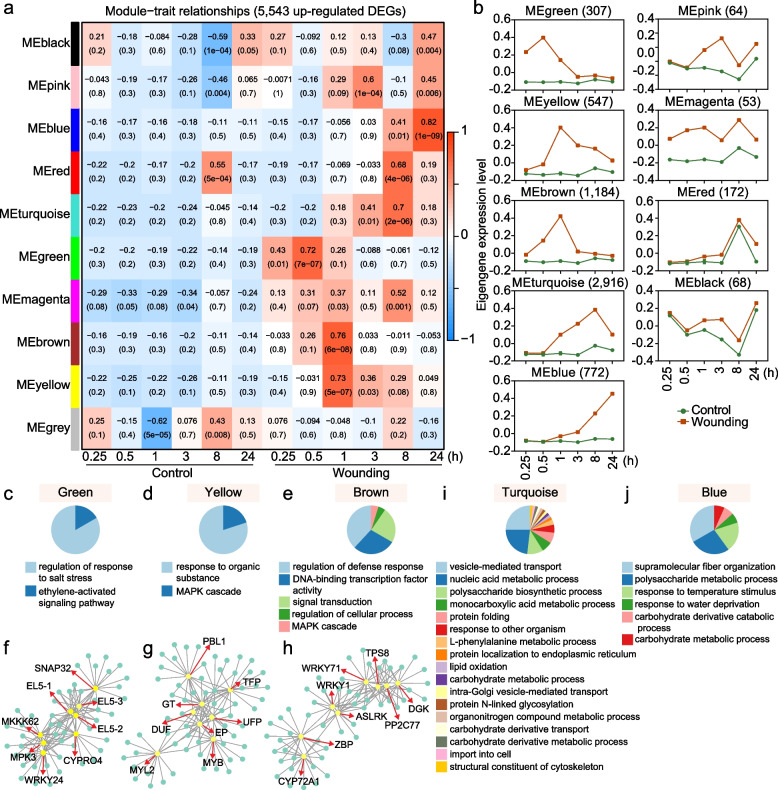
Identification of early and late wound-induced up-regulated gene modules by weighted gene co-expression network analysis (WGCNA). **a** Heatmap showing the module-trait correlations. Gene with FC > 2, FDR value < 0.05 at least one time point and FC > −2 across all time points was defined as up-regulated DEG. The numbers in the heatmap show the correlation (upper) and the P-values (lower). **b** Eigengene expression pattern for each identified module. The number of genes in each module was given. **c-e** Gene ontology (GO) analysis of genes in the three early modules (green, yellow and brown) by ClueGO. GO terms (*P* < 0.05) with similar biological functions were grouped and the most significant term in each group was shown. The percentage of each functional group corresponds with the number of terms included in the group. **f–h** Hub genes (yellow circle) of “green” (f), “yellow” (g) and “brown” (h) modules. Eight genes in each module with the top module membership (MM) were defined as hub genes. For ease of visualization, eight hub genes and up to fifteen of their neighbors based on the sort of edge weight are shown. The weight of the edge connecting each hub gene and its neighbors was at least 0.25. i-j GO analysis of genes in the two late modules (turquoise and blue) by ClueGO. GO terms (P < 0.05) with similar biological functions were grouped and the most significant term in each group was shown. The percentage of each functional group corresponds with the number of terms included in the group

To better understand expression patterns of wound-responsive genes, we performed weighted gene co-expression network analysis (WGCNA) using 5,543 wound-induced upregulated DEGs. This analysis identified nine co-expressed gene modules with different expression patterns (Fig. 3a and b; Table S2). Among them, the green, yellow, and brown modules contained early-responsive genes (peaking at ≤ 1 hpw), while the turquoise and blue modules represented late-responsive genes (peak peaking at ≥ 8 hpw). The green module genes were rapidly induced, peaking at 0.5 hpw, while genes in the yellow and brown modules peaked at 1 hpw. These early-responsive genes were enriched in terms related to regulators, e.g., regulation of abiotic stresses, mitogen-activated protein kinase (MAPK) cascade, regulation of defense response, DNA-binding transcription factor activity, and signaling transduction (Fig. 3c-e). Several kinases (*MPK3*, *MKKK62*, *DGK*, *ASLRK*) and transcription factors (*WRKY24*, *MYL2*, *MYB*, *WRKY1*, *WRKY71*) were identified as hub genes within these modules (Fig. 3f-h). In contrast, the turquoise and blue modules represented late-responsive genes, peaking after 8 hpw. These genes were preferentially involved in transport processes (e.g., vesicle-mediated transport, carbohydrate derivative transport), cell wall components biosynthesis and organization (e.g. polysaccharide biosynthetic process, structural constituent of cytoskeleton, supramolecular fiber organization), and specialized metabolites biosynthesis, e.g., L-phenylalanine metabolic processes, monocarboxylic acid metabolic processes, lipid oxidation (Fig. 3i and j).

### Transcription factors, kinases, phytohormones, and peptides are key wound-responsive regulators

**Fig. 4:**
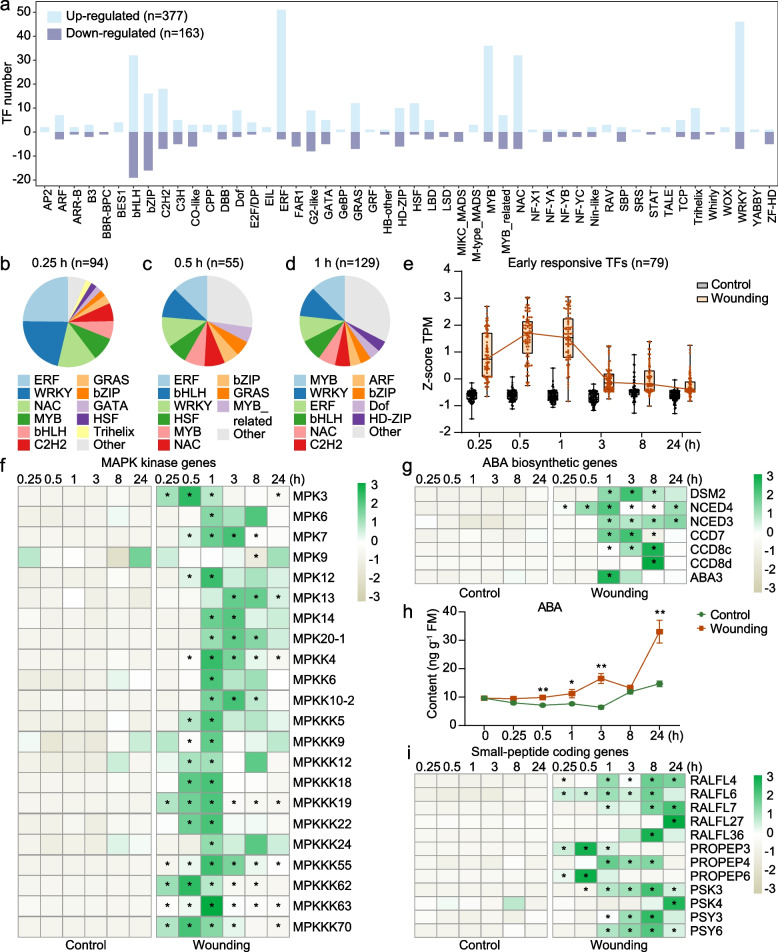
Early responsive regulators in rice leaves upon wounding treatment. **a** Number of up-regulated and down-regulated transcription factors (TFs) responsive to wounding. **b-d** Composition of early responsive TFs at 0.25 h, 0.5 h and 1 h after wounding. **e** Box plots showing the total relative expression levels of TFs which started to be up-regulated at 0.25 h after wounding and peaked within 1 h treatment (*n* = 79) in RNA-seq data. In each box plot, the horizontal bar in the box indicates the median value. The upper and lower hinges of each box indicate the 75% and 25% ranges of the reported values, respectively. **f-g** Relative transcript levels of MAPK kinase genes (f) and abscisic acid (ABA) biosynthetic genes (g) in wounded and control leaves. Numbers in the color key indicate the row z-score of the TPM value in RNA-seq data. Asterisks indicate significant differences between different treatments at each time point (*, FC > 2 and FDR < 0.05). **h** Mean concentrations (± SE, *n* = 5) of ABA in wounded and control leaves. Asterisks indicate significant differences between different treatments at each time point (*, P < 0.05; **, P < 0.01; Student’s t test). **i** Relative transcript levels of small-peptide coding genes in wounded and control leaves. Numbers in the color key indicate the row z-score of the TPM value in RNA-seq data. Asterisks indicate significant differences between different treatments at each time point (*, FC > 2 and FDR < 0.05)

We next dissected putative genes involved in some early- and late-responsive pathways. Transcription factors (TFs) play a central role in transcriptional regulation, and our transcriptome analysis identified 377 upregulated and 163 downregulated TF genes across 50 TF families that were significantly induced by wounding (Fig. 4a; Table S3). Among them, early responsive TFs, i.e. 0.25-1 hpw, were predominantly from the ERF, WRKY, NAC, MYB, and bHLH TF families (Fig. 4b-d). A total of 79 TFs were rapidly upregulated at 0.25 hpw, reaching peak expression levels at 0.5 hpw (Fig. 4e), including well-documented regulators of rice resistance to herbivores, such as ERF3, WRKY53, and WRKY71 [38–40]. Protein kinases, crucial for early signaling transduction via phosphorylation [41, 42], were also significantly induced within 1 h of wounding (Fig. S2a). In addition to MAPK cascade genes, calcium/calmodulin-dependent protein kinase (*CAMK*) genes were abundant among upregulated kinases (Fig. 4f; Fig. S2b).

Given the enrichment of early wound-responsive genes in plant response to salt stress, we evaluated the involvement of the abscisic acid (ABA) signaling pathway, a key regulator of abiotic stress responses [43, 44]. Core ABA biosynthetic genes were significantly upregulated within 1 hpw, with the 9-*cis*-epoxycarotenoid dioxygenase (*NCED4*) gene showing rapid induction at 0.25 hpw (Fig. 4g). Consistently, ABA level was significantly elevated in wounded leaves as early as 0.5 hpw, suggesting its role in early wound responses (Fig. 4h).

A growing body of evidence has highlighted the role of small peptides-triggered signaling in plant wounding responses [5–7, 45, 46]. In agreement with this, we identified several wound-induced small peptide-encoding genes, including five rapid alkalization factors (*RALF*), three plant elicitor peptide precursors (*PROPEP*), two phytosulfokines (*PSK*), and two peptide containing sulfated tyrosine (*PSY*) were identified (Fig. 4i). Notably, the transcript levels of *RALF4*, *RALF6*, *PROPEP3* and *PROPEP6* were rapidly increased from 0.25 hpw. Taken together, these results suggest that besides JA signaling, many TFs, small peptides, kinases and the ABA signaling pathway may also contribute to the regulation of wound responses in rice.

### Wounding affects phenylpropanoid metabolism

**Fig. 5:**
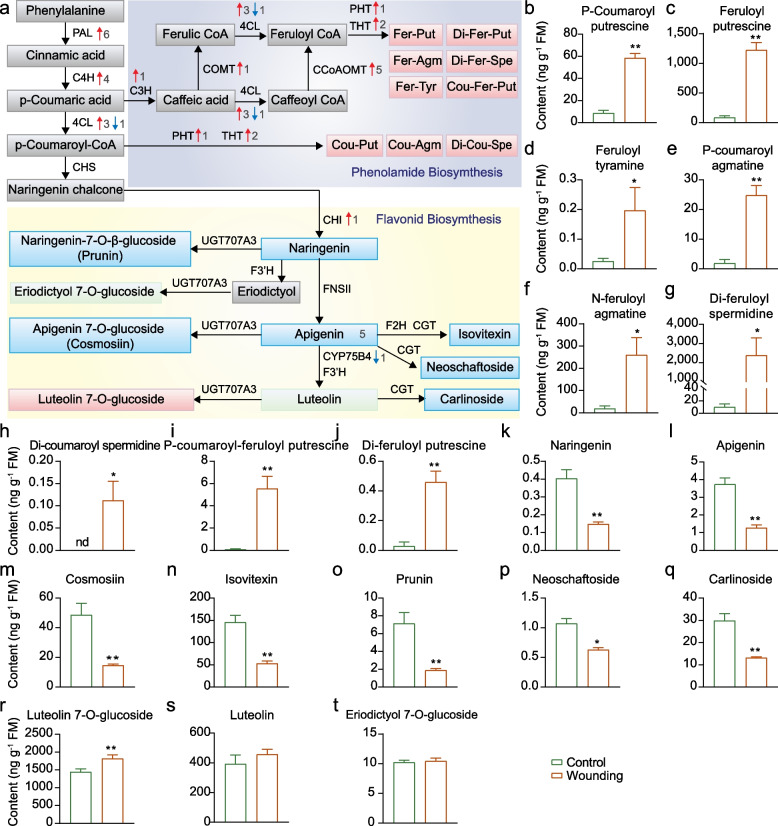
The biosynthesis of phenolamides and flavonoids in rice leaves upon wounding treatment. **a** Expression patterns of phenolamides and flavonoids biosynthetic genes upon wounding treatment. The red and blue arrows represent genes that are up- and down-regulated, respectively. The number beside the arrow represents the number of DEGs. For metabolites, the red box represents wound-induced up-accumulated metabolites, the blue box represents down-regulated metabolites, and the green box represents metabolite levels that are not significantly changed. **b-t** Mean levels (+ SE, *n* = 5) of nine phenolamides (b-j) and ten flavanoids (k-t) in rice leaves 24 h after wounding treatment. Leaves of non-treated plants were used as controls. Asterisks indicate significant differences in wounded leaves compared with control leaves (*, *P* < 0.05; **, *P* < 0.01; Student’s t test)

Based on the above GO enrichment analysis (Fig. 3i), we investigated late responsive genes related to L-phenylalanine metabolic process i.e. the phenylpropanoid pathway. Key genes involved in the initial steps of this pathway, including six phenylalanine ammonia-lyase genes (*PAL*), four cinnamate-4-hydroxylase genes (*C4H*), and three 4-coumarate:CoA ligase genes (*4CL*) genes, were significantly upregulated upon wounding (Fig. 5a). To identify which phenylpropanoid derivatives were activated upon wounding, we analyzed the expression of downstream biosynthetic genes. Most phenolamide biosynthetic genes, including various acyltransferase genes, putrescine hydroxycinnamoyl acyltransferase (*PHT4*), and tryptamine/tyramine hydroxycinnamoyl transferase (*THT1*, *THT2*), were significantly upregulated (Fig. 5a). Consistently, the concentration of nine phenolamides was significantly increased in wounded leaves (Fig. 5b-j). In contrast, the flavonoid biosynthetic genes showed no significant differences between wounded and control leaves (Fig. 5a). However, metabolite analysis revealed a general decline in flavonoid levels, except for luteolin-7-*O*-glucoside which increased, while eriodictyol-7-*O*-glucoside and luteolin remained unchanged (Fig. 5k-t). These results suggest that wounding promotes phenolamides accumulation while suppressing flavonoids biosynthesis.

### Transcriptional changes induced by leaf folder (LF) feeding differ from mechanical wounding

**Fig. 6:**
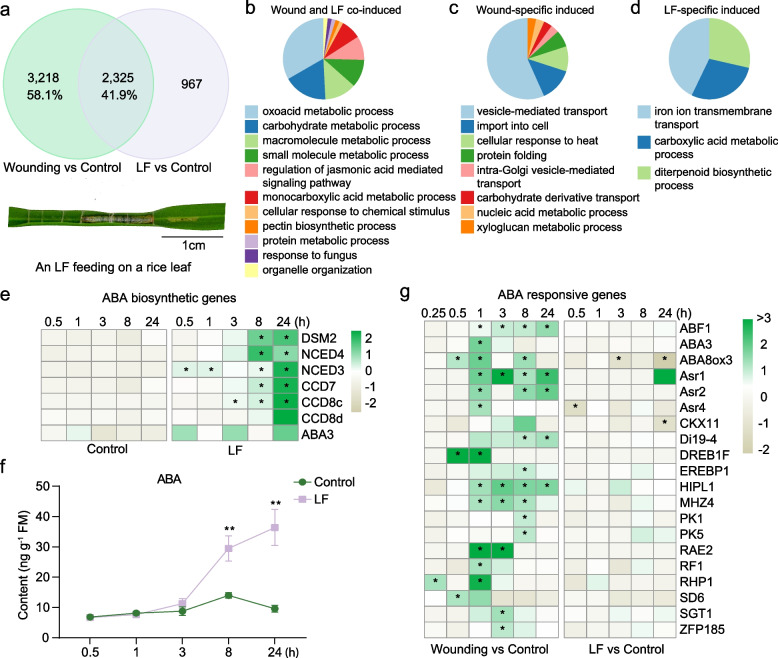
Common and specific transcriptional changes induced by leaf folder (LF) and wounding treatment. **a** Venn diagram showing the number of up-regulated DEGs induced by wounding and LF infestation. Gene with FC > 2, FDR value < 0.05 at least one time point and FC > −2 across all time points was defined as up-regulated DEG. The photograph below the Venn shows an LF feeding on a rice leaf. Scale bar, 1 cm. **b-d** Gene ontology (GO) analysis of wound and LF co-induced (**b**), wound-specific induced (**c**) and LF-specific induced genes (**d**) by ClueGO. GO terms (P < 0.05) with similar biological functions were grouped and the most significant term in each group was shown. The percentage of each functional group corresponds with the number of terms included in the group. **e** Relative transcript levels of ABA biosynthetic genes in LF-treated and untreated control leaves. Numbers in the color key indicate the row z-score of the TPM value in RNA-seq data. Asterisks indicate significant differences in LF-damaged leaves compared with untreated control leaves (*, FC > 2 and FDR < 0.05). **f** Mean concentrations (± SE, *n* = 6) of ABA in rice leaves under LF infestation. Leaves of non-treated plants at each time point were used as controls. Asterisks indicate significant differences in LF-treated leaves compared with untreated control leaves at each time point (**, *P* < 0.01; Student’s t test). **g** Relative expression changes of ABA responsive genes in wounded and LF-treated leaves compared with their corresponding controls. Numbers in the color key indicate the log_2_FC (fold change) in wounded or LF-treated leaves compared to control leaves from the RNA-seq data. Asterisks indicate significant differences between different treatments at each time point (*, |FC|> 2 and FDR < 0.05)

Leaf folder (LF) is a major insect pest in the paddy field that damages plants by rolling rice leaves and scraping cells of the upper epidermis and mesophyll tissues. The RNA-seq analysis of LF-induced responses was performed as previously described [47]. Leaf samples were collected at 0.5, 1, 3, 8, and 24 h post-LF treatment, with untreated plant leaves collected at corresponding time points serving as controls. To evaluate the correlation between LF- and wound-induced transcriptional responses, we compared upregulated DEGs from both treatments (Fig. 6a). Only 41.9% of wound-induced upregulated DEGs were also responsive to LF feeding, suggesting that LF suppresses certain wound-related responses in rice (Fig. 6a). GO analysis revealed that genes co-induced by LF feeding and wounding were enriched in pathways related to the regulation of jasmonic acid mediated signaling pathway and specialized metabolism (Fig. 6b). In contrast, wound-specific induced DEGs were associated with transport processes and cellular response to heat (Fig. 6c), while LF-specific induced DEGs were preferentially involved in specialized metabolite biosynthesis (Fig. 6d). The activation of JA signaling by LF feeding has been investigated previously [47]. We further examined other early signaling events. Fewer small-peptide coding genes and kinase genes were upregulated by LF compared to mechanical wounding (Fig. 4i; Fig. S2; Fig. S3a-c). However, certain genes, such as small-peptide coding genes (e.g. *RALF22*) and kinase genes (e.g. *CDPK5*, *CPK18*) were specially induced by LF feeding. Similar to mechanical wounding, LF feeding also triggered ABA biosynthesis (Fig. 6e-f). However, many ABA-responsive genes upregulated by wounding were not induced by LF feeding (Fig. 6g), suggesting that LF feeding actively suppresses some wound responses.

## Discussion

Wound responses and their regulation have been extensively studied in dicots, particularly in *Arabidopsis* [1, 48]. However, our understanding of these processes in the important crop plant rice remains limited. In this study, we investigated wound-induced transcriptional changes in rice leaves and identified key molecular components involved in this response. First, we observed dynamic gene expression changes even in untreated plants over the course of a day, highlighting the importance of using time-matched controls when identifying DEGs. Second, we found that JA and JA-Ile were rapidly accumulated in leaves upon wounding, followed by the late accumulation of their derivatives, indicating that JA biosynthesis and catabolism are strongly wound-responsive and very likely play a central role in regulating any kind of wound-related responses in rice. Third, we identified several early wound-responsive components, including ERF and WRKY transcription factors, MAPK kinases, RALF and PROPEP peptides, and ABA biosynthesis. In contrast, the biosynthesis of specialized metabolites was late responsive. Two phenylpropanoid derivatives, phenolamides and flavonoids, exhibited opposite accumulation patterns in response to wounding. Lastly, while LF larvae feeding causes several wounds to rice leaves, it induces a distinct transcriptional response compared to mechanical wounding, with certain wounding responses, such as ABA-mediated downstream pathway, being suppressed by LF infestation.

A PCA analysis of the transcriptome data showed that samples collected at dusk (8 h after wounding) clustered separately from those collected at other time points, suggesting that time of the day (i.e. the circadian rhythm of the plant) significantly contributes to transcriptional variation in addition to the applied stress. This phenomenon has also been reported in previous studies [36, 47, 49, 50]. When comparing gene expressions across control samples collected at different time points, we identified a substantial number of DEGs, with the highest number found in samples collected at 8 h compared to others. Therefore, using only 0 hpw samples as controls for treatment comparisons can lead to false identification of DEGs. For instance, transcript levels of circadian clock genes, *LUX ARRYTHMO* (*LUX*) and *CIRCADIAN CLOCK ASSOCIATED 1* (*CCA1*), were highest and lowest, respectively, in control samples collected at 8 h compared with other timepoints (Fig. S4a and b). However, their expression did not significantly differ between wound-treated and control samples collected at the same time point, which strongly suggests their wound-independent expression pattern. This study, along with our previous transcriptome data of brown planthopper-, methyl jasmoanate-, and LF-treatment, provides strong evidence that multiple time-matched controls are essential when investigating time-series stress-induced transcriptional responses to ensure the accurate identification of truly relevant DEGs.

Upon mechanical wounding, the JA biosynthetic pathway was rapidly activated in rice, with the JA precursor OPDA, JA itself and the bioactive conjugate JA-Ile accumulating and peaking within 1 hpw. Subsequently, the JA catabolic pathway was induced, with hydroxylation of JA and JA-Ile reaching their respective peak at 8 hpw and 3 hpw (Fig. 2). Although JA plays a crucial role in plant defense against abiotic and biotic stressors, its accumulation often comes at the expense of plant growth, a phenomenon known as growth-defense tradeoff [18, 51–53]. Wounded plants typically exhibit reduced growth following JA induction [51, 53]. To prevent JA signaling running out of control, plants have evolved negative feedback regulation mechanisms, including JA catabolism [18, 25]. P450 cytochromes of the CYP94 subfamily convert JA-Ile into its hydroxylated and carboxylated forms, 12-OH-JA-Ile and 12-COOH-JA-Ile, respectively [54, 55]. Additionally, JA oxidases (JOX) oxidize JA into 12-OH-JA, effectively restraining JA-Ile production. Mutations of three *JOX*s in *Arabidopsis* lead to a retarded growth compared to wild-type plants [56, 57]. Thus, when JA reaches a threshold in wounded rice plants, the JA catabolic pathway is engaged to maintain an optical balance between defense and growth.

Phenylpropanoid metabolism was strongly activated in response to mechanical wounding in rice. Interestingly, two major phenylpropanoid derivatives, phenolamides and flavonoids, exhibited opposed regulatory patterns. Most of phenolamides were significantly accumulated in wounded leaves, while the levels of most flavonoids were reduced. A similar contrasting pattern of phenylpropanoid levels was also observed in LF-treated plants [58]. The wound-induced expression of phenolamide biosynthetic genes was consistent with their increased accumulation. However, the expression of flavonoid biosynthetic genes remained unchanged, suggesting that the decreased flavonoid levels may result from a shift in metabolic flux within the phenylpropanoid pathway during wounding. The biosynthesis of phenolamides or other unidentified phenylpropanoids may competitively consume common substrates, such as *p*-coumaric acid, thereby reducing flavonoid production. A similar trend has been reported previously, where overexpression of *EgMYB4* from oil palm in sweet basil suppressed lignin biosynthesis while enhancing the accumulation of methyleugenol and methylchavicol, both of which share a common precursor with lignin [59].

By comparing wound- and LF-induced transcriptomes, we found that many genes upregulated by mechanical wounding did not respond to LF larva feeding (Fig. 6a). The difference between the two treatments is that LF larva releases oral secretions (OS) into wounds during feeding. Some elicitors or effectors from OS can either activate or suppress plant wound-induced defense responses [60–63]. In the OS of cotton bollworm (*Helicoverpa armigera*), HARP1 and HAS1 have been identified as effector proteins that suppress JA-induced defense in both *Arabidopsis* and cotton [60, 61]. This suggests that similar effectors may exist in LF OS. We found that the expression of most ABA-responsive genes was significantly upregulated by wounding, while their expression remained unchanged after LF feeding (Fig. 6g). ABA has been implicated in plant defense against herbivores [64, 65]. For instance, overexpression of ABA biosynthetic gene *OsNCED3* in rice enhances plant resistance to BPH [64]. Moreover, in *Nicotiana attenuata*, NaHER1 positively contributes to herbivore resistance by suppressing ABA catabolism [65]. The suppression of ABA-responsive genes suggests a potential adaptation strategy used by LF to impair rice defense responses.

In summary, this study provides a comprehensive analysis of wound-induced transcriptional changes in rice. The identification of early- and late-responsive pathways and genes will help in dissecting the defense-related signaling network in rice. Moreover, these data are valuable resources for future investigation studies on rice and herbivore/pathogen interactions.

## Materials and methods

### Plant materials and growth conditions

The japonica rice cultivars (*O. sativa* subsp. *japonica*) Xiushui 11 (XS11) were used. Seeds were germinated with water in Petri dishes in an illuminated incubator at 27 ± 1 °C under 16 h of light. After 7 days, seedlings were transferred into hydroponic cultivation and grown in a growth chamber under 14 h light (28 °C) and 10 h dark (26 °C) photoperiod as described previously [51]. Plants were used for experiments after 21 to 25 days.

### Plant treatments and sample collections

For wounding treatment, the youngest fully expanded leaf was wounded by rolling a fabric pattern wheel along both sides of the midvein, generating two parallel rows of evenly distributed small punctures (each ~ 20 cm in length). Samples were harvested at six time points (0.25, 0.5, 1, 3, 8, and 24 h. Leaves from untreated plants at each time point served as controls. The samples were frozen in liquid nitrogen and stored at − 80 °C.

### RNA-seq analysis

Total RNA was isolated from control and wounded rice leaves. RNA-seq was performed by Novogene. (https://www.novogene.com/). Three biological replicates were used for each treatment. Adaptor sequences and low-quality reads were removed using Fastp (version 0.23.4) [66]. Clean reads were mapped to the reference rice genome (http://rice.plantbiology.msu.edu/pub/data/Eukaryotic_Projects/o_sativa/annotation_dbs/ pseudomolecules/) using Hisat2 (version 2.2.1) [67]. The read counts were obtained and normalized (transcripts per million, TPM) using Stringtie (version 2.2.1) [68]. PCA was performed using the R package GGORD. DEGs were analyzed using the R package edgeR (version 3.38.4) [69]. Genes with FC > 2, FDR value < 0.05 across all six time points were defined as upregulated DEGs. GO enrichment analysis was performed using ClueGO [70]. The significance of the GO term is calculated using a two-sided hypergeometric test. Then, the Bonferroni step-down and Kappa score = 0.4 were used for the *P*-value correction.

### Weighted gene co-expression network analysis (WGCNA)

All identified wound-induced upregulated DEGs (5,543) were used for WGCNA using the R package WGCNA (version 1.71), with the following settings: soft-thresholding power (β) = 10, minimum module size = 30, and branch merge cut height = 0.25. Genes with no significant network correlations were assigned to the gray module. Eight genes in each module with the top module membership (MM) were defined as hub genes. The network was drawn using Cytoscape [71].

### Phytohormone analyses

Approximately 50 mg of leaf material (precise mass was recorded) was ground and extracted with 800 µL ethyl acetate containing the internal standards (20 ng of D6-JA, 5 ng of D6-JA-Ile and 5 ng of D6-ABA). Extracts were analyzed by LC–MS 8040 (Shimazu, Kyoto, Japan) as described previously [58].

### Phenolamides and flavonoids measurements

Approximately 85 mg of leaf material (precise mass was recorded) was ground and extracted twice with 800 and 500 µL 70% methanol. The supernatants were combined and then evaporated using a nitrogen blower to remove methanol. The remaining water was freeze-dried using a vacuum freeze dryer. The dried sample was dissolved with 130 µL 70% methanol and analyzed via liquid chromatography-mass spectrometry with an electrospray ionization source (Agilent 6460). The content of each compound was calculated by the standard curve method. Five biological replicates per treatment were analyzed.

### Data analysis

Student’s t test was used to compare the differences between different treatments at each time point (*, p < 0.05; **, p < 0.01; Student’s t test) using DPS software.

## Supplementary Information

Supplementary Material 1.

Supplementary Material 2.

## Data Availability

The RNA-seq data reported in this paper have been deposited in the Genome Sequence Archive at the BIG Data Center (http://bigd.big.ac.cn/gsa), Beijing Institute of Genomics (BIG), Chinese Academy of Sciences, under accession number CRA023554 (wound-related data) and CRA004405 (LF-related data).
