# Supplementary material for: Wound-induced transcriptional dynamics in rice
Source: Crop Health. 2025 Jul 1;3(1):15. doi: 10.1007/s44297-025-00055-2 (PMC12825920; doi:10.1007/s44297-025-00055-2)
Supplement: Supplementary file 1 — Supplementary Material 1. [file 44297_2025_55_MOESM1_ESM.docx]

**Supplementary Information**


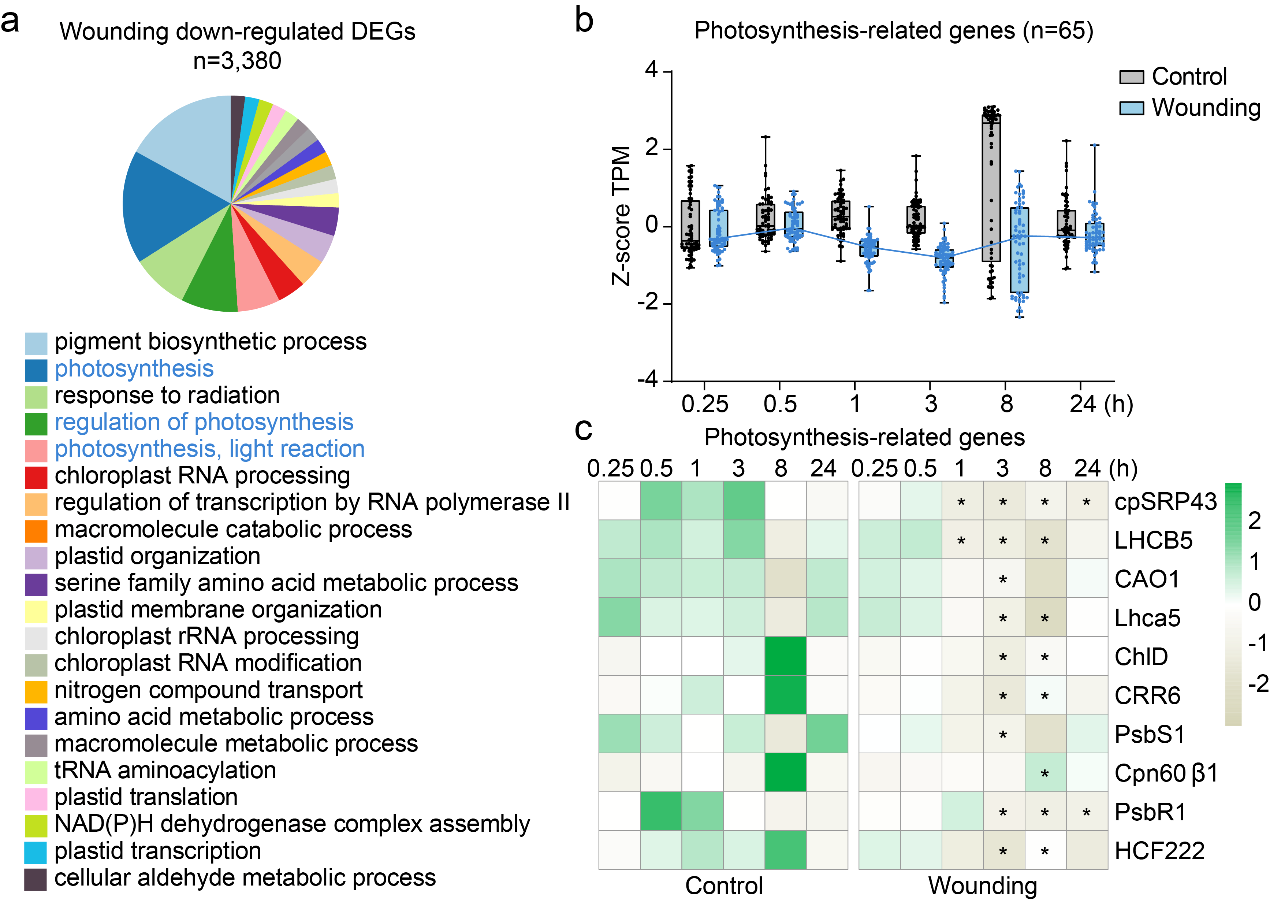


**Fig. S1** Wounding impairs rice photosynthesis. **a** Gene ontology (GO) analysis of down-regulated DEGs upon wounding treatment by ClueGO. Gene with FC < -2, FDR value < 0.05 at least one time point and FC < 2 across all time points was defined as down-regulated DEG. GO terms (P < 0.05) with similar biological functions were grouped and the most significant term in each group was shown. The percentage of each functional group corresponds with the number of terms included in the group. Terms related to photosynthesis are marked in blue. **b** Box plots showing the total relative expression level of photosynthesis-related genes (n = 65) upon wounding treatment in RNA-seq data. In each box plot, the horizontal bar in the box indicates the median value. The upper and lower hinges of each box indicate the 75% and 25% ranges of the reported values, respectively. **c** Relative transcript levels of several photosynthesis-related genes in wounded and control leaves. Numbers in the color key indicate the row z-score of the TPM value in RNA-seq data. Asterisks indicate significant differences between different treatments at each time point (*, FC < -2 and FDR < 0.05)


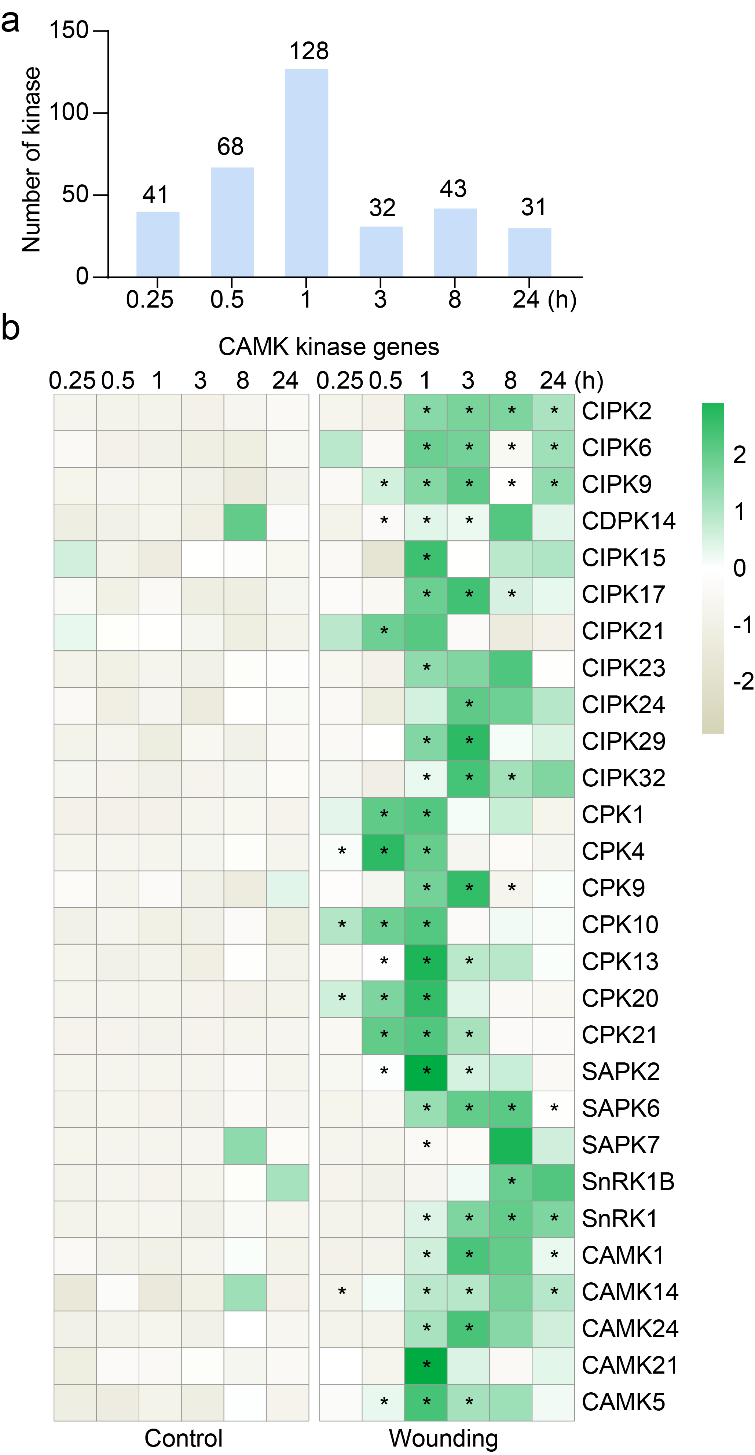


**Fig. S2** Wound-induced kinase genes in rice leaves. **a** Number of up-regulated kinase DEGs in wounded leaves compared with control leaves. **b** Relative transcript levels of CAMK kinase genes in wounded and control leaves. Numbers in the color key indicate the row z-score of the TPM value in RNA-seq data. Asterisks indicate significant differences between different treatments at each time point (*, FC > 2 and FDR < 0.05).


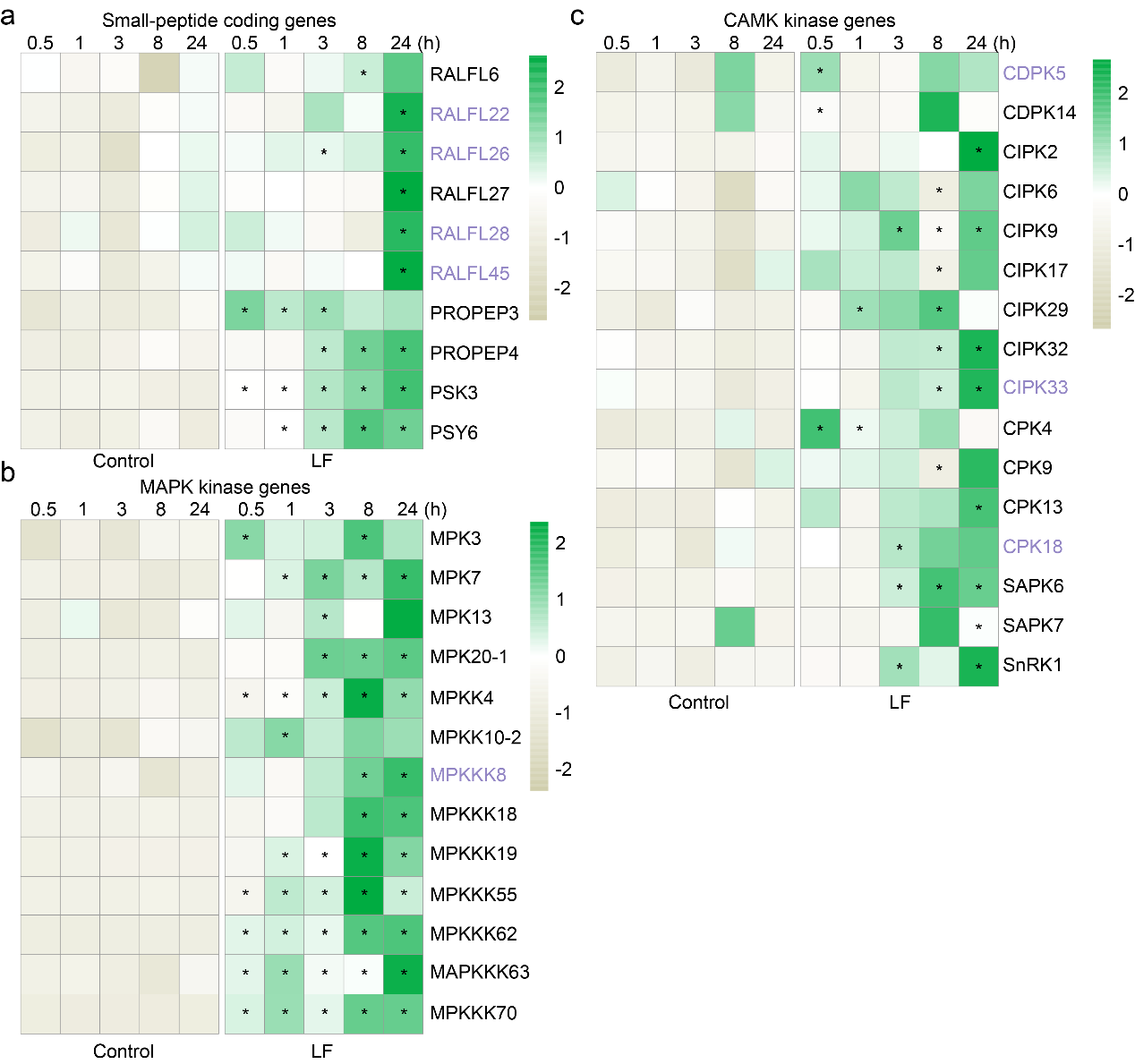


**Fig. S3** Relative transcript levels of small-peptide coding genes (**a**), MAPK kinase genes (**b**) and CAMK kinase genes (**c**) in LF-treated and control leaves without any treatment. Numbers in the color key indicate the row z-score of the TPM value in RNA-seq data. Asterisks indicate significant differences in LF-treated leaves compared with untreated-control leaves at each time point (*, FC > 2 and FDR < 0.05). LF-specific induced genes compared with wound induced genes were highlighted in purple.


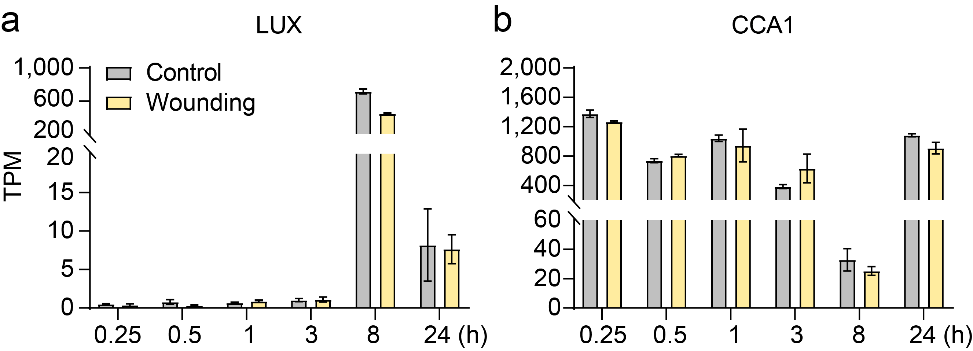


**Fig. S4** Relative transcript levels of two circadian clock genes in rice leaves upon wounding treatment. Mean TPM values (± SE, n = 3) of *LUX ARRYTHMO* (*LUX*, **a**) and *CIRCADIAN CLOCK ASSOCIATED 1* (*CCA1*, **b**) in wound-treated and control leaves by RNA-seq.
